# Association of Vitamin D Deficiency With Cardiometabolic and Renal Outcomes in Patients With Autoimmune Rheumatic Diseases: A Systematic Review and Meta-Analysis

**DOI:** 10.7759/cureus.113623

**Published:** 2026-07-29

**Authors:** Adnan Imran, Hafiz Abdul Waris, Sikander Khan Tanoli, Muhammad Tabish Raza, Kamran Memon, Hassan Saeed

**Affiliations:** 1 Acute Medicine, Walsall Manor Hospital, Walsall, GBR; 2 Cardiology, Walsall Manor Hospital, Walsall, GBR; 3 Acute Medicine, University Hospitals Coventry and Warwickshire NHS Trust, Coventry, GBR; 4 Internal Medicine, Jahra Hospital, Al Jahra, KWT; 5 Cardiac Emergency, Armed Forces Institute of Cardiology and National Institute of Heart Diseases, Rawalpindi, PAK

**Keywords:** autoimmune rheumatic disorders, cardiometabolic risk, renal outcomes, rheumatoid arthritis, systemic lupus erythematosus, vitamin d deficiency

## Abstract

Autoimmune rheumatic diseases (ARDs) are chronic immune-mediated diseases associated with increased cardiovascular and renal morbidity. Vitamin D deficiency is common in these disorders and may coexist with inflammation, immune dysregulation, endothelial dysfunction, and impaired vascular and renal homeostasis. This systematic review and meta-analysis evaluated the association of vitamin D deficiency with adverse cardiometabolic and renal outcomes in ARDs. PubMed/MEDLINE, Scopus, Embase, Web of Science, and Google Scholar were searched from inception through March 31, 2026, without language restrictions. Observational studies that measured serum 25-hydroxyvitamin D (25(OH)D) and reported cardiometabolic, cardiovascular, renal, or cardiorenal outcomes were eligible. Two reviewers independently conducted study selection, data extraction, and quality assessment. Compatible outcome-specific estimates were pooled using constrained maximum-likelihood random-effects models with Hartung-Knapp inference. Hazard ratios (HRs) and correlation coefficients were examined independently. Eighteen studies met the eligibility criteria, and four independent cohorts contributed to at least one quantitative synthesis. Vitamin D deficiency was associated with higher all-cause mortality (pooled HR 1.95, 95% confidence interval (CI) 1.36-2.79). The pooled association with cardiovascular events was imprecise (HR 2.20, 95% CI 0.52-9.25). Serum 25(OH)D was inversely but inconclusively correlated with proteinuria in pediatric systemic lupus erythematosus (r = -0.47, 95% CI −0.98 to 0.85). Cardiometabolic studies generally reported adverse metabolic and vascular findings but could not be pooled because of incompatible effect measures. Vitamin D deficiency was associated with higher mortality and greater cardiometabolic and renal disease burden; however, cardiovascular-event and proteinuria estimates remained inconclusive. Observational designs, residual confounding, heterogeneous definitions, and few compatible studies precluded causal conclusions. Prospective studies and randomized trials are required to determine whether correcting vitamin D deficiency improves clinical outcomes.

## Introduction and background

Autoimmune rheumatic diseases (ARDs), such as rheumatoid arthritis, systemic lupus erythematosus (SLE), Sjögren syndrome, systemic sclerosis, and associated connective tissue diseases, are persistent immune-mediated conditions that may affect the joints, blood vessels, kidneys, and several other organs [1]. In addition to musculoskeletal manifestations, ARDs are associated with persistent systemic inflammation and an increased burden of cardiovascular, metabolic, and renal complications [2,3]. Cardiovascular morbidity is particularly important in rheumatoid arthritis and SLE, whereas immune-mediated renal injury remains a major determinant of prognosis in patients with lupus nephritis [3,4].

Inflammation, oxidative stress, immune dysregulation, and endothelial injury may contribute to these complications. Endothelial dysfunction and arterial stiffness are early manifestations of vascular damage, while sustained immune activation may promote insulin resistance, vascular remodeling, and progressive organ dysfunction [3,5]. In rheumatoid arthritis, observational studies have linked low serum 25-hydroxyvitamin D (25(OH)D) concentrations with metabolic syndrome, dyslipidemia, endothelial dysfunction, and arterial stiffness [6-9]. Studies involving patients with SLE have similarly reported associations between vitamin D deficiency and disease activity, proteinuria, vascular stiffness, metabolic syndrome, and insulin resistance [10-12].

Vitamin D has biological functions extending beyond calcium and bone metabolism. Through the vitamin D receptor, it contributes to immune tolerance, T-cell differentiation, B-cell regulation, and inflammatory cytokine signaling [13,14]. Vitamin D receptors are expressed in immune cells, vascular endothelial and smooth muscle cells, cardiomyocytes, and renal tissue. These observations provide biological plausibility for associations between vitamin D deficiency, systemic inflammation, endothelial dysfunction, oxidative stress, and altered renin-angiotensin-aldosterone system activity [5,13,15]. Nevertheless, biological plausibility does not establish that vitamin D deficiency directly causes cardiovascular or renal complications.

Vitamin D deficiency is frequently reported in patients with ARDs and may be influenced by limited sunlight exposure, photosensitivity, reduced physical activity, obesity, dietary factors, glucocorticoid therapy, and inflammatory disease activity [1,16]. Serum 25(OH)D, the principal indicator of vitamin D status, is produced through hepatic 25-hydroxylation. In contrast, renal 1α-hydroxylation converts 25(OH)D into the active metabolite 1,25-dihydroxyvitamin D. Impaired renal hydroxylation therefore primarily reduces calcitriol concentrations and should not be described as a principal direct cause of low serum 25(OH)D. Reverse causation and residual confounding must also be considered because disease activity, obesity, renal dysfunction, treatment exposure, and reduced outdoor activity may influence both vitamin D status and cardiorenal risk. Furthermore, current evidence does not establish that vitamin D supplementation prevents cardiovascular or renal events in patients with ARDs [17,18].

Individual studies have reported associations between vitamin D deficiency and cardiometabolic abnormalities, proteinuria, lupus-related disease activity, and major cardiovascular and kidney events [10,19]. However, the evidence remains limited by small sample sizes, heterogeneous adult and pediatric populations, inconsistent definitions of vitamin D deficiency, varied outcome measures, and differences in the adjustment for confounding. Pediatric findings may not be directly comparable with adult outcomes because disease duration, clinical course, and the timing of complications differ between these populations [10,20]. Therefore, this systematic review and meta-analysis aimed to evaluate the association of vitamin D deficiency with adverse cardiometabolic and renal outcomes in patients with ARDs and to assess the consistency and certainty of the available evidence.

## Review

Materials and methods

Study Design and Reporting

This systematic review and meta-analysis assessed the association between vitamin D insufficiency and adverse cardiometabolic and renal outcomes in individuals with ARDs. The review was conducted and reported in accordance with the Preferred Reporting Items for Systematic Reviews and Meta-Analyses (PRISMA) 2020 guidelines. Eligible populations were adults and children diagnosed with specific autoimmune rheumatic conditions; the exposure assessed was vitamin D deficiency, indicated by serum 25(OH)D levels; and the comparisons involved adequate or elevated vitamin D levels.

Protocol and Registration

The review protocol was not prospectively registered. Eligibility criteria, outcome definitions, data-extraction procedures, and analytical decisions were therefore reported in sufficient detail to improve transparency and reproducibility.

Literature Search

PubMed/MEDLINE, Scopus, Embase, Web of Science, and Google Scholar were examined from their inception until March 31, 2026. Controlled vocabulary and free-text terms about vitamin D, autoimmune rheumatic conditions, cardiometabolic risk, cardiovascular ailments, and renal outcomes were combined using “AND” and “OR”.

The PubMed/MEDLINE strategy was (“vitamin D deficiency” OR “25-hydroxyvitamin D” OR “25(OH)D” OR “vitamin D status”) AND (“autoimmune rheumatic disease” OR “rheumatoid arthritis” OR “systemic lupus erythematosus” OR “Sjögren syndrome” OR “psoriatic arthritis” OR “ankylosing spondylitis” OR “systemic sclerosis” OR “inflammatory myopathy” OR “juvenile dermatomyositis”) AND (“cardiovascular disease” OR “cardiometabolic risk” OR “metabolic syndrome” OR “insulin resistance” OR “dyslipidaemia” OR “endothelial dysfunction” OR “arterial stiffness” OR “hypertension” OR “proteinuria” OR “renal disease” OR “chronic kidney disease” OR “major adverse cardiovascular” OR “major adverse kidney events” OR “mortality”).

The strategy was adapted for each database. Reference lists of eligible studies and relevant reviews were screened for additional records. No language restriction was applied. Non-English studies were assessed using translated abstracts and full texts where available. Complete database-specific strategies and search yields were recorded for the supplementary material.

Eligibility Criteria

Cross-sectional, case-control, and cohort studies were eligible if they included patients with an ARD, measured serum 25(OH)D, defined or quantified vitamin D deficiency, and reported at least one cardiometabolic, cardiovascular, renal, or cardiorenal outcome. Adult and pediatric studies were eligible for the qualitative synthesis, but their findings were analyzed separately where differences in disease duration or outcome timing limited comparability.

Studies were included in the quantitative synthesis only when they reported a compatible effect estimate with a measure of uncertainty or provided sufficient raw data to perform a valid calculation. Case reports, case series, incomplete conference abstracts, editorials, letters, reviews, meta-analyses, laboratory or animal studies, duplicate cohorts, and studies without relevant or extractable data were excluded. Studies lacking a reproducible quantitative estimate were retained in the narrative synthesis but excluded from pooling.

Study Selection

All identified records were transferred into reference-management software, and redundancies were eliminated. Two evaluators independently examined titles and abstracts before evaluating the full texts deemed potentially eligible. Conflicts were resolved through discussion and consensus; no outstanding disputes persisted after the consensus evaluation. The rationale for the complete-text exclusion was documented, and the selection methodology was illustrated in a PRISMA 2020 flowchart.

Data Extraction

Two reviewers independently extracted data using a piloted standardized form. Extracted information included author, year, country, design, rheumatic disorder, sample size, age group, sex distribution, disease duration, vitamin D assay, serum 25(OH)D threshold, comparator group, outcome definition, follow-up duration, effect measure, confidence interval (CI), adjustment variables, and raw numerical data used for effect-size calculation.

Adjusted estimates were preferred when available, and the model accounting for the most relevant confounders was selected. Every quantitative estimate was classified as directly reported or calculated from published data. ORs, HRs, risk ratios, correlations, and mean differences were not considered interchangeable. Extraction disagreements were resolved through consensus.

Quality and Risk-of-Bias Assessment

Two reviewers independently assessed cohort and case-control studies using the Newcastle-Ottawa Scale. An adapted version was used for cross-sectional studies. The assessment covered participant selection, comparability, exposure measurement, outcome assessment, and, where applicable, adequacy of follow-up. Scores of 7-9 indicated high quality, 5-6 moderate quality, and less than 5 low quality. Studies were not excluded solely based on their quality score; risk-of-bias findings informed sensitivity analyses and certainty-of-evidence judgments.

Outcome Classification

Cardiometabolic outcomes included metabolic syndrome, insulin resistance, dyslipidemia, endothelial dysfunction, arterial stiffness, and validated cardiovascular-risk biomarkers. Renal outcomes included proteinuria, lupus nephritis or renal disease activity, chronic kidney disease progression, and major adverse kidney events. Major adverse cardiovascular events and all-cause mortality were evaluated as separate clinical outcomes.

Clinically dissimilar endpoints were not combined into a single composite analysis. In particular, proteinuria, renal disease activity, major adverse kidney events, major adverse cardiovascular events, and mortality were analyzed separately unless an original study reported them as a predefined composite outcome.

Effect-Size Derivation

Directly reported odds ratios (ORs) and hazard ratios (HRs) were transformed to the natural logarithmic scale. Their standard errors were calculated as $\frac{\ln(\text{upper }95\%~\mathrm{CI}) - \ln(\text{lower }95\%~\mathrm{CI})}{3.92}$. For 2 × 2 tables, ORs were calculated as $\frac{a \times d}{b \times c}$, with the standard error of ln(OR) calculated as $\sqrt{\frac{1}{a} + \frac{1}{b} + \frac{1}{c} + \frac{1}{d}}$. A 0.5 continuity correction was applied only when a cell contained zero. HRs were analyzed separately and were not converted to ORs.

Correlation coefficients were transformed using Fisher’s $z = \frac{1}{2}\ln\!\left(\frac{1+r}{1-r}\right)$, with variance $\frac{1}{n - 3}$, and pooled values were back-transformed to the correlation scale. Estimates were not derived from narrative findings, p-values alone, or figures without reliable numerical data. A supplementary effect-derivation table documented the source statistic, calculation method, variance, adjustment status, and inclusion decision for each estimate.

Statistical Analysis

Analyses were conducted in R using the meta package (R Foundation for Statistical Computing, Vienna, Austria). Meta-analysis was performed only when at least two independent studies reported sufficiently comparable exposures, outcomes, and effect measures. ORs and HRs were synthesized in separate models. Random-effects meta-analysis was conducted using restricted maximum likelihood estimation of between-study variance. The Hartung-Knapp adjustment was used for pooled confidence intervals because few studies were expected in each analysis. Common-effect estimates were reported only as sensitivity analyses.

Only one estimate from each independent cohort was included in a given primary outcome analysis. When a study reported multiple clinically distinct outcomes, these were entered into separate outcome-specific models. Multiple Chen et al. endpoints were not treated as independent estimates in one pooled analysis. Publications derived from the overlapping Systemic Lupus International Collaborating Clinics cohort were retained only for distinct outcomes; overlapping estimates for the same outcome were not pooled.

Heterogeneity was evaluated using Cochran’s Q, $I^2$, and $\tau^2$. Approximate $I^2$ values of 0-25%, 26-50%, 51-75%, and greater than 75% represented low, moderate, substantial, and considerable heterogeneity, respectively. These thresholds were interpreted with caution because estimates of heterogeneity are imprecise when few studies are included. Meta-regression was not performed because fewer than 10 independent studies were available per outcome.

Sensitivity and Subgroup Analyses

Sensitivity analyses compared common-effect and random-effects estimates and excluded studies with high risk of bias, unadjusted estimates, or overlapping cohorts. Where sufficient data were available, subgroup or sensitivity analyses examined adult versus pediatric populations, rheumatic disease subtypes, study designs, vitamin D thresholds, and adjusted versus unadjusted estimates. Analyses were not performed when fewer than two independent studies were available within a subgroup.

Publication Bias

Publication bias and small-study effects were only informally tested when fewer than 10 independent studies contributed to an analysis. Any funnel plots presented under these circumstances were considered exploratory and were not interpreted as evidence of the presence or absence of publication bias. Egger’s regression was not performed because of insufficient statistical power.

Certainty of Evidence

Certainty was assessed individually for each clinically specified outcome utilizing the Grading of Recommendations Assessment, Development and Evaluation framework. Observational evidence was initially assigned a low-certainty rating and evaluated for risk of bias, inconsistency, indirectness, imprecision, and publication bias. The possibility of enhancement was considered for a significant impact, an exposure-response correlation, or residual confounding that could diminish the detected association. The evaluations were documented in a summary-of-findings table tailored to specific outcomes.

Ethical Considerations

This analysis used aggregated data from previously published research and did not involve patient enrolment, intervention, or access to identifiable personal information. Institutional ethics approval and informed consent were consequently unnecessary.

Results

Study Selection

The updated literature search yielded 1,309 records, including 1,284 records retrieved from electronic databases and 25 records identified through citation searching and reference-list screening. Two additional eligible reports were identified during the updated search. After removal of 312 duplicate records, 997 titles and abstracts were screened, of which 921 were excluded. Seventy-six full-text reports were retrieved and assessed for eligibility. Fifty-eight reports were excluded because of an ineligible population or vitamin D exposure, an ineligible outcome or study design, an overlapping cohort, or insufficient extractable outcome data. No report was excluded solely because of publication language.

**Figure 1:**
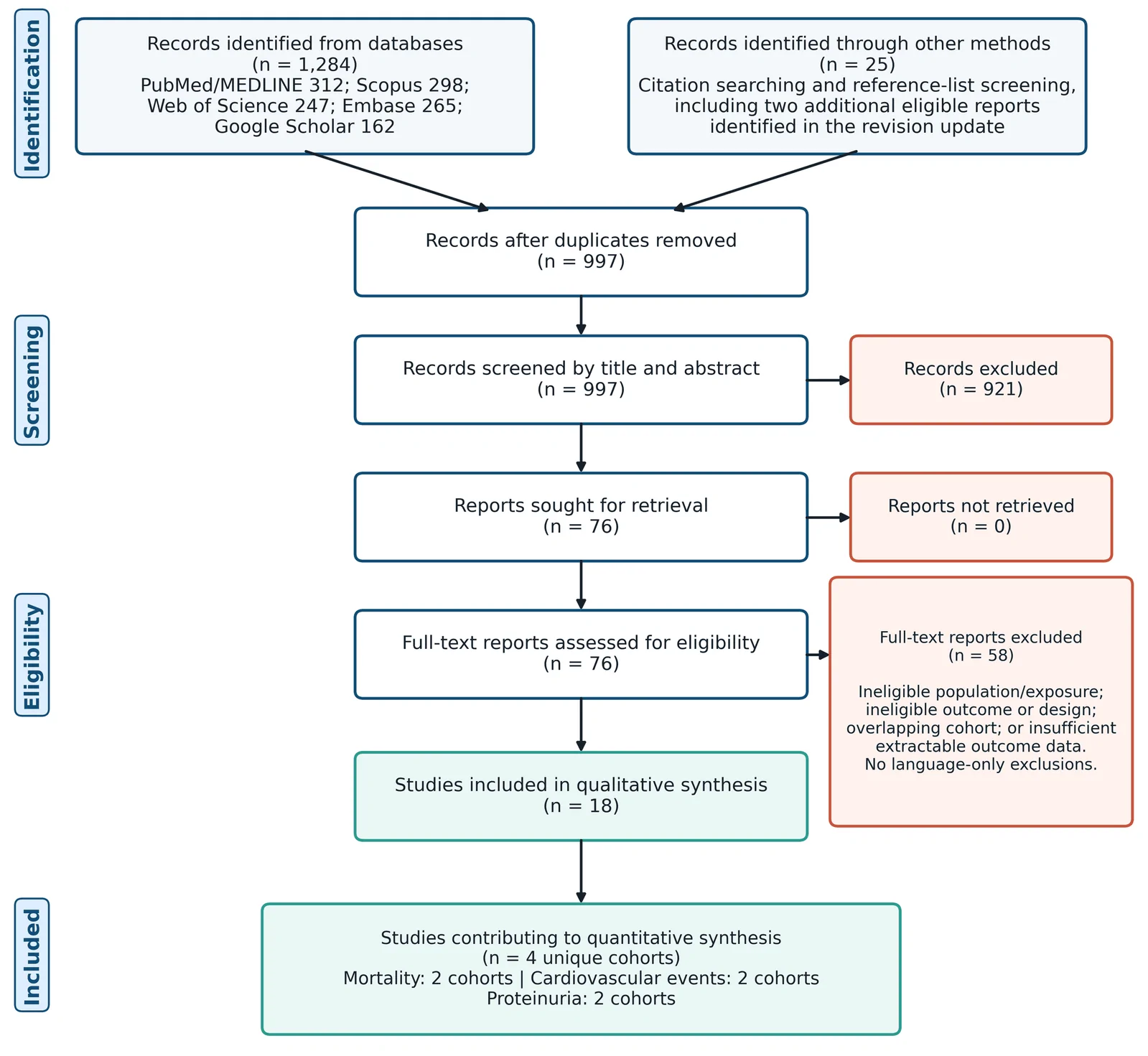
PRISMA 2020 flowchart illustrating the revised study-selection methodology A cumulative total of 1,309 records was discovered. After eliminating duplicates, screening, and comprehensive text evaluation, 18 studies were included in the qualitative synthesis. Four independent cohorts contributed to at least one quantitative synthesis. No report was excluded solely because of publication language. PRISMA: Preferred Reporting Items for Systematic Reviews and Meta-Analyses

Eighteen studies were included in the qualitative synthesis. Four independent cohorts contributed to at least one quantitative synthesis. Two cohorts contributed to the mortality analysis, two contributed to the cardiovascular-event analysis, and two pediatric SLE cohorts contributed to the proteinuria analysis. Because individual cohorts contributed to more than one outcome domain, cohort counts were not added across analyses. The complete study-selection process is presented in Figure 1.

Study Characteristics and Overall Evidence Base

The 18 included studies comprised six studies of rheumatoid arthritis, 10 studies of SLE or juvenile dermatomyositis, and two studies of primary Sjögren syndrome. Most studies used cross-sectional or case-control designs. Chen et al. conducted a large retrospective propensity-score-matched cohort study, whereas Assawasaksakul et al. analyzed prospective data from the Hopkins Lupus Cohort.

Chew et al. and Lertratanakul et al. analyzed overlapping populations from the multinational Systemic Lupus International Collaborating Clinics inception cohort. Consequently, their participant numbers were not treated as independent, and their estimates were not entered simultaneously into the same outcome analysis.

Serum 25(OH)D was the principal exposure. Vitamin D deficiency was generally defined as a serum 25(OH)D concentration below 20 ng/mL, whereas insufficiency was commonly defined using a threshold below 30 ng/mL. However, the thresholds varied among studies. Therefore, “lower vitamin D status” is used as the general descriptive term when summarizing studies with different exposure definitions.

Several studies analyzed serum 25(OH)D as a continuous exposure rather than applying a binary deficiency threshold. Rivera-Escoto et al. additionally assessed calcidiol, calcitriol, soluble vitamin D receptor, and the calcitriol-to-calcidiol ratio. Findings from these alternative vitamin D biomarkers were included in the qualitative synthesis but were not pooled with estimates from binary 25(OH)D deficiency.

The outcomes evaluated included metabolic syndrome, insulin resistance, dyslipidemia, endothelial dysfunction, arterial stiffness, cardiovascular risk biomarkers, cardiovascular events, proteinuria, lupus nephritis, renal disease activity, major adverse kidney events, mortality, and rheumatic disease activity. The characteristics and principal findings of the included studies are summarized in Table 1.

**Table 1:** Characteristics and principal findings of the included studies 25(OH)D: 25-hydroxyvitamin D, BMI: body mass index, CI: confidence interval, CVD: cardiovascular disease, HDL: high-density lipoprotein, HOMA-IR: homeostatic model assessment of insulin resistance, HR: hazard ratio, IMT: intima-media thickness, JDM: juvenile dermatomyositis, MACE: major adverse cardiovascular events, MAKE: major adverse kidney events, NOS: Newcastle-Ottawa Scale, OR: odds ratio, RA: rheumatoid arthritis, S/C/O: selection/comparability/outcome, SLE: systemic lupus erythematosus, SLICC: Systemic Lupus International Collaborating Clinics

Study	Country and design	Population	Vitamin D assessment	Outcomes	Principal source-verified finding	Synthesis	NOS S/C/O; total; quality
Haque et al., 2012 [6]	USA; cross-sectional	RA, n = 179	Serum 25(OH)D; <30 ng/mL	HDL, HOMA-IR, fibrinogen and endothelial biomarkers	Lower 25(OH)D was associated with an adverse metabolic and endothelial profile; no compatible binary effect estimate was available	Cardiometabolic; narrative	3/2/3; 8; high
Delgado-Frías et al., 2015 [7]	Spain; case-control	RA, n = 111; controls, n = 105	Circulating vitamin D; continuous	Flow-mediated dilation and osteoprotegerin	No clear independent association with endothelial dysfunction	Endothelial; narrative	3/2/3; 8; high
Baker et al., 2012 [8]	USA; cross-sectional	RA, n = 499	Deficiency <20 ng/mL	Metabolic syndrome and hyperlipidemia	Metabolic syndrome: adjusted OR 3.45 (95% CI 1.75-6.80); hyperlipidemia: OR 1.72 (95% CI 1.10-2.45)	Individual ORs; not pooled	3/2/3; 8; high
Lo Gullo et al., 2015 [9]	Italy; case-control	RA, n = 27; controls, n = 41	Continuous serum 25(OH)D	Inflammation, arterial stiffness, carotid measures and CD34+ cells	Mixed inflammatory and vascular correlations; no vitamin D-deficiency OR	Vascular; narrative	3/1/3; 7; high
Robinson et al., 2012 [10]	USA; cross-sectional	Pediatric SLE, n = 37; JDM, n = 21	Continuous serum 25(OH)D	Proteinuria and disease activity	In pediatric SLE, serum 25(OH)D correlated inversely with urinary protein/creatinine, r=−0.60	Proteinuria meta-analysis	3/2/3; 8; high
Reynolds et al., 2012 [11]	UK; cross-sectional	Women with SLE, n = 75	Deficiency <20 ng/mL	Aortic stiffness, carotid IMT and plaque	Vitamin D deficiency was independently associated with increased aortic stiffness	Vascular; narrative	3/2/3; 8; high
Chew et al., 2021 [12]	Multinational; inception-cohort analysis	SLE, n = 1,163	Continuous and grouped 25(OH)D	Metabolic syndrome and insulin resistance	Lower vitamin D was associated with metabolic syndrome and insulin resistance	Narrative; overlapping SLICC cohort	4/2/3; 9; high
Chen et al., 2026 [19]	Multinational TriNetX; retrospective matched cohort	Sjögren syndrome with osteoporosis; 1,067 matched pairs	<20 versus ≥30 ng/mL	Mortality, MACE, MAKE and fractures	Mortality HR 1.92; MACE HR 2.11; MAKE HR 2.34	Mortality and cardiovascular-event meta-analyses; MAKE reported separately	4/2/3; 9; high
Lin et al., 2018 [20]	Taiwan; case-cohort	Pediatric-onset SLE, n = 35	Continuous 25(OH)D	Disease activity, nephritis and proteinuria	Lower vitamin D was associated with greater disease activity and renal involvement	Renal; narrative	3/1/2; 6; moderate
Rivera-Escoto et al., 2024 [21]	Mexico; case-control	Women with RA, n = 154; controls, n = 201	Vitamin D metabolites and hydroxylation ratio	Cardiovascular-risk classification	Selected calcitriol measures were associated with cardiovascular-risk classification but did not provide a binary 25(OH)D-deficiency estimate	Cardiovascular; narrative	3/2/2; 7; high
Caraba et al., 2017 [22]	Romania; case-control	Early RA, n = 35; controls, n = 35	Continuous serum 25(OH)D	Disease activity, insulin resistance and endothelial function	Lower vitamin D correlated with greater disease activity, insulin resistance, and endothelial dysfunction	Cardiometabolic; narrative	3/1/2; 6; moderate
Attar and Siddiqui, 2013 [23]	Saudi Arabia; cross-sectional	SLE, n = 95	Deficiency <20 ng/mL	Disease activity, proteinuria and renal involvement	Lower vitamin D was associated with greater disease activity and renal involvement	Renal; narrative	3/1/2; 6; moderate
Wu et al., 2009 [24]	USA; cross-sectional	Women with SLE, n = 181	Continuous and categorical 25(OH)D	BMI, blood pressure, lipids and inflammation	Selected cardiovascular-risk associations were attenuated after adjustment for BMI	Cardiometabolic; narrative	3/2/3; 8; high
Mok et al., 2012 [25]	Hong Kong; cross-sectional	SLE, n = 290	Insufficiency <30 and deficiency <15 ng/mL	Disease activity, vascular risk and atherosclerosis	Lower vitamin D was associated with disease activity and dyslipidemia but not subclinical atherosclerosis	Cardiometabolic; narrative	3/2/3; 8; high
Lertratanakul et al., 2014 [26]	Multinational; inception-cohort analysis	SLE, n = 890	Continuous baseline 25(OH)D	Cardiovascular-risk profile and incident CVD	Higher vitamin D was associated with favorable risk profiles; evidence for incident CVD was limited	Narrative; overlapping SLICC cohort	4/2/3; 9; high
Zardi et al., 2016 [27]	Italy; case-control	Primary Sjögren syndrome, n = 25; controls, n = 22	Continuous serum 25(OH)D	Carotid IMT, disease activity and damage	Vitamin D was unrelated to IMT but inversely associated with disease activity and damage	Cardiovascular and disease activity; narrative	3/1/2; 6; moderate
Jiang et al., 2023 [28]	China; retrospective	Childhood-onset SLE, n = 168	<12, 12-<20 and ≥20 ng/mL; continuous analysis	Lupus nephritis and 24-hour urinary protein	Serum 25(OH)D correlated inversely with 24-hour urinary protein, r = −0.384	Proteinuria meta-analysis	3/2/2; 7; high
Assawasaksakul et al., 2026 [29]	USA; prospective cohort	SLE, n = 1,768	<20 versus 30-39 ng/mL	Mortality and cardiovascular events	Mortality HR 2.05; cardiovascular-event HR 2.98	Mortality and cardiovascular-event meta-analyses	4/2/3; 9; high

Distribution of Evidence Across Outcome Domains

Lower vitamin D status was associated with at least one adverse outcome in most included studies, although the direction and strength of the associations varied across endpoints. Adverse findings were most frequent for metabolic abnormalities and endothelial dysfunction in rheumatoid arthritis, disease activity and renal involvement in SLE, and mortality or cardiovascular events in the longitudinal Sjögren syndrome and SLE cohorts.

Mixed or endpoint-specific findings were common across studies assessing cardiovascular risk biomarkers, arterial stiffness, and subclinical atherosclerosis. Delgado-Frías et al. did not identify a clear independent association with endothelial dysfunction. Mok et al. identified associations with dyslipidemia and disease activity, but not with subclinical atherosclerosis, whereas Zardi et al. found no association with carotid intima-media thickness.

The distribution and direction of findings across the included studies are summarized in Table 2. Correlations, regression coefficients, ORs, and HRs were not converted into a single common measure for this evidence map.

**Table 2:** Evidence map of the 18 included studies A: adverse association between lower vitamin D status and the assessed outcome, M: mixed or endpoint-specific findings, N: no clear independent association, -: the outcome domain was not assessed

Study	Metabolic/insulin	Endothelial/arterial	CV events	Renal/proteinuria	Activity/inflammation	Mortality
Haque et al., 2012 [6]	A	A	-	-	A	-
Delgado-Frías et al., 2015 [7]	-	N	-	-	-	-
Baker et al., 2012 [8]	A	-	-	-	-	-
Lo Gullo et al., 2015 [9]	-	M	M	-	M	-
Robinson et al., 2012 [10]	-	-	-	A	A	-
Reynolds et al., 2012 [11]	-	A	A	-	-	-
Chew et al., 2021 [12]	A	-	-	-	-	-
Chen et al., 2026 [19]	-	-	A	A	-	A
Lin et al., 2018 [20]	-	-	-	A	A	-
Rivera-Escoto et al., 2024 [21]	-	-	M	-	M	-
Caraba et al., 2017 [22]	A	A	-	-	A	-
Attar and Siddiqui, 2013 [23]	-	-	-	A	A	-
Wu et al., 2009 [24]	M	-	M	-	M	-
Mok et al., 2012 [25]	A	N	N	-	A	-
Lertratanakul et al., 2014 [26]	M	-	M	-	A	-
Zardi et al., 2016 [27]	-	N	M	-	A	-
Jiang et al., 2023 [28]	-	-	-	A	A	-
Assawasaksakul et al., 2026 [29]	-	-	A	-	-	A

Methodological Quality and Risk of Bias

Fourteen studies were classified as high quality and four as moderate quality; none was classified as low quality. The strongest methodological ratings were assigned to the longitudinal cohort and large inception-cohort analyses. Moderate-quality ratings were assigned primarily to small cross-sectional or case-control studies with limited adjustment for confounding.

The principal risk-of-bias concerns were cross-sectional temporality, small or single-center samples, heterogeneous vitamin D thresholds, differences in vitamin D assays, incomplete adjustment for confounding, and selective reporting of multiple correlated endpoints. Relevant potential confounders included age, sex, obesity, sunlight exposure, ethnicity, vitamin D supplementation, disease activity, corticosteroid and immunosuppressive treatment, renal function, and pre-existing cardiovascular disease.

Although the longitudinal cohorts reduced concerns regarding temporality, residual confounding and exposure misclassification remained possible. No study was excluded solely because of its methodological quality rating.

Cardiometabolic Outcomes

The cardiometabolic studies could not be combined into a common OR meta-analysis because they reported statistically incompatible effect measures. Baker et al. reported adjusted ORs for binary metabolic outcomes, whereas the remaining studies predominantly reported correlations, continuous regression coefficients, group comparisons, or associations involving alternative vitamin D metabolites.

Baker et al. found that vitamin D deficiency was associated with metabolic syndrome in rheumatoid arthritis, with an adjusted OR of 3.45 (95% CI 1.75-6.80). The corresponding OR for hyperlipidemia was 1.72 (95% CI 1.10-2.45).

Haque et al. reported that lower 25(OH)D was associated with an adverse cardiometabolic and endothelial biomarker profile in rheumatoid arthritis. Caraba et al. observed inverse relationships between 25(OH)D and insulin resistance, disease activity, and endothelial function. Chew et al. found that lower vitamin D was associated with metabolic syndrome and insulin resistance in SLE. Reynolds et al. reported an independent association between vitamin D deficiency and increased aortic stiffness.

Conversely, Delgado-Frías et al. found no clear independent relationship with endothelial dysfunction. Mok et al. observed an association with dyslipidemia but not with subclinical atherosclerosis, whereas Zardi et al. found no association with carotid intima-media thickness. The cardiometabolic findings were therefore synthesized narratively, and no pooled cardiometabolic OR was calculated.

All-Cause Mortality

Two independent longitudinal cohorts reported adjusted HRs for all-cause mortality using comparable deficient-versus-reference 25(OH)D categories. Chen et al. reported an HR of 1.92 (95% CI 1.41-2.62), while Assawasaksakul et al. reported an HR of 2.05 (95% CI 1.19-3.54).

**Figure 2:**
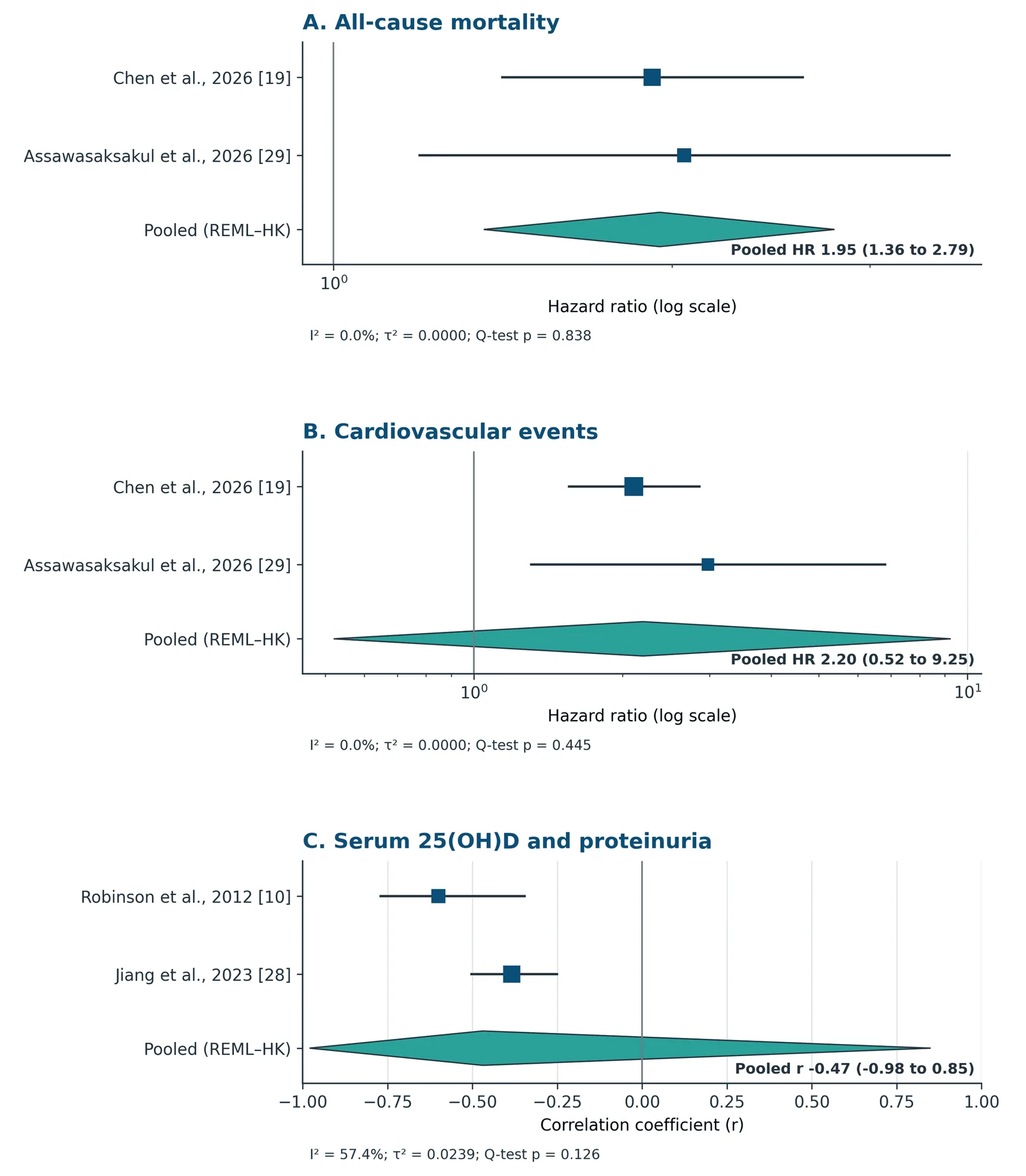
Forest plots of the source-verifiable quantitative syntheses (A) Adjusted HRs for all-cause mortality. (B) Adjusted HRs for cardiovascular events. (C) Correlations between serum 25(OH)D and proteinuria. HRs were pooled only with HRs. Correlation coefficients were Fisher-z transformed before pooling and subsequently back-transformed. HRs: hazard ratios, 25(OH)D: 25-hydroxyvitamin D

The random-effects analysis produced a pooled HR of 1.95 (95% CI 1.36-2.79), indicating higher all-cause mortality among participants with vitamin D deficiency. No statistical heterogeneity was detected (I² = 0.0%, τ² = 0; Cochran Q p = 0.838). Nevertheless, the heterogeneity estimate should be interpreted cautiously because only two independent cohorts were available. The study-level and pooled estimates are presented in Figure 2A and Table 3.

**Table 3:** Quantitative meta-analysis and sensitivity-analysis results CI: confidence interval, HR: hazard ratio, SLE: systemic lupus erythematosus

Outcome domain	Studies	Effect measure	Random-effects estimate	I²	τ²	Cochrane Q p-value	Common-effect estimate
All-cause mortality	Two independent cohorts	HR	1.95 (95% CI 1.36-2.79)	0.0%	0	0.838	1.95 (95% CI 1.49-2.55)
Cardiovascular events	Two independent cohorts	HR	2.20 (95% CI 0.52-9.25)	0.0%	0	0.445	2.20 (95% CI 1.65-2.94)
Proteinuria	Two independent pediatric SLE cohorts	Correlation coefficient	−0.47 (95% CI −0.98 to 0.85)	57.4%	0.0239	0.126	−0.43 (95% CI −0.53 to −0.31)

Cardiovascular Events

Two independent longitudinal cohorts contributed adjusted cardiovascular-event HRs. Chen et al. reported an HR of 2.11 (95% CI 1.55-2.88), while Assawasaksakul et al. reported an HR of 2.98 (95% CI 1.30-6.85).

The pooled random-effects HR was 2.20 (95% CI 0.52-9.25). No statistical heterogeneity was detected (I² = 0.0%, τ² = 0; Cochran Q p = 0.445). Although the point estimate suggested a higher cardiovascular event rate among participants with vitamin D deficiency, the Hartung-Knapp CI was wide and included the null value. The finding should therefore be interpreted as suggestive rather than conclusive.

Chen et al.’s major adverse kidney-event estimate was not entered into the cardiovascular-event analysis because it represented a separate clinical outcome. The cardiovascular-event analysis is presented in Figure 2B and Table 3.

Renal and Proteinuria Outcomes

Two pediatric SLE cohorts reported compatible correlation coefficients between serum 25(OH)D and urinary protein. Robinson et al. reported an inverse correlation between serum 25(OH)D and urinary protein/creatinine in 37 children with SLE (r = -0.60). Jiang et al. reported an inverse correlation between serum 25(OH)D and 24-hour urinary protein in 168 children with initial-onset SLE (r = -0.384).

The pooled correlation was r = -0.47 (95% CI −0.98 to 0.85), with moderate statistical heterogeneity (I² = 57.4%, τ² = 0.0239; Cochran Q, p = 0.126). The direction of the pooled estimate suggested that lower serum 25(OH)D was associated with greater proteinuria. However, the CI was extremely wide and included the null because only two studies were available.

Pediatric estimates were analyzed separately from adult mortality and cardiovascular outcomes because age, disease duration, and the timing of renal and cardiovascular complications could modify the observed relationships. Dichotomous lupus-nephritis findings were retained in the narrative synthesis because compatible adjusted binary estimates were not consistently available.

Chen et al. separately reported an HR of 2.34 (95% CI 1.69-3.24) for major adverse kidney events. This time-to-event estimate was not pooled with cross-sectional correlations for proteinuria. The proteinuria meta-analysis is presented in Figure 2C and Table 3.

Sensitivity Analysis

**Figure 3:**
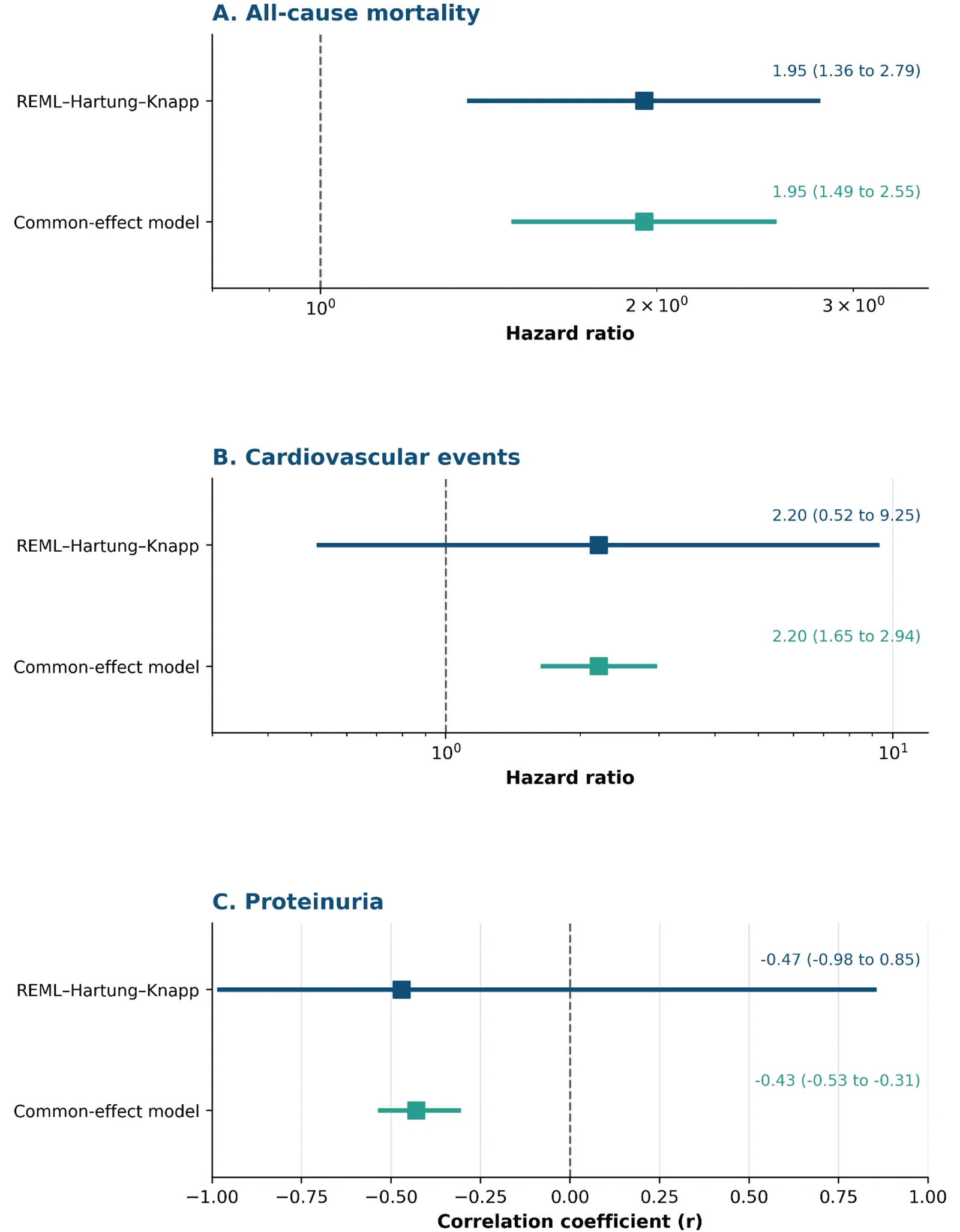
Sensitivity analysis comparing the random-effects and common-effect models Random-effects estimates were calculated using restricted maximum likelihood with Hartung-Knapp inference. Point estimates were similar across models, but common-effect CIs were narrower because they did not incorporate between-study and small-sample uncertainty. CIs: confidence intervals

The pooled point estimates remained stable when random-effects and common-effect models were compared, although the common-effect CIs were substantially narrower. For all-cause mortality, the random-effects model yielded an HR of 1.95 (95% CI 1.36-2.79), whereas the common-effects model yielded an HR of 1.95 (95% CI 1.49-2.55). For cardiovascular events, the random-effects model yielded an HR of 2.20 (95% CI 0.52-9.25), while the common-effects model yielded an HR of 2.20 (95% CI 1.65-2.94). For proteinuria, the random-effects model yielded a pooled correlation of r = -0.47 (95% CI −0.98 to 0.85), whereas the common-effects model yielded r = -0.43 (95% CI −0.53 to −0.31). The difference in confidence-interval width reflected the incorporation of small-sample uncertainty in the Hartung-Knapp analysis. Leave-one-out analyses were not informative because each quantitative synthesis contained only two independent studies. The quantitative and sensitivity-analysis results are summarized in Table 3 and Figure 3.

Publication Bias and Small-Study Effects

Funnel plots and formal tests for funnel-plot asymmetry were not performed because each quantitative synthesis contained only two independent studies. With substantially fewer than 10 studies, visual funnel-plot assessment and regression-based asymmetry tests would have insufficient power to reliably distinguish publication bias from chance. Publication bias and small-study effects therefore could not be excluded.

Certainty of Evidence

The certainty of evidence was rated as low for all-cause mortality and very low for cardiovascular events, proteinuria, and metabolic syndrome. The outcome-specific certainty ratings and reasons for downgrading are presented in Table 4. The mortality evidence was downgraded because it originated from observational studies and was based on only two cohorts. Cardiovascular-event evidence was downgraded due to observational design and substantial imprecision, as the Hartung-Knapp CI was wide and included the null.

**Table 4:** GRADE-style certainty assessment CI: confidence interval, HR: hazard ratio, OR: odds ratio, RA: rheumatoid arthritis

Outcome	Evidence base	Principal effect estimate	Certainty	Main reasons for rating
All-cause mortality	Two longitudinal cohorts [19,29]	HR 1.95 (95% CI 1.36-2.79)	Low	Observational evidence and serious imprecision
Cardiovascular events	Two longitudinal cohorts [19,29]	HR 2.20 (95% CI 0.52-9.25)	Very low	Observational evidence and very serious imprecision
Proteinuria	Two pediatric cross-sectional cohorts [10,28]	r = −0.47 (95% CI −0.98 to 0.85)	Very low	Cross-sectional evidence, heterogeneity, pediatric indirectness and very serious imprecision
Metabolic syndrome in RA	One cross-sectional study [8]	OR 3.45 (95% CI 1.75-6.80)	Very low	Single observational study, residual confounding and inability to assess consistency

Evidence for proteinuria was downgraded due to cross-sectional measurement, moderate heterogeneity, restriction to pediatric SLE populations, and very serious imprecision. Evidence for metabolic syndrome was based on a single cross-sectional study of rheumatoid arthritis and could not be pooled. These ratings apply to the certainty of the reported associations. They do not establish a causal effect of vitamin D deficiency or demonstrate that vitamin D supplementation prevents cardiometabolic or renal outcomes.

Discussion

This systematic review and meta-analysis evaluated cardiometabolic and renal outcomes associated with vitamin D deficiency across ARDs. The quantitative synthesis showed that the available evidence could not be summarized using a single pooled OR because the included studies used different vitamin D exposures, clinical endpoints, and effect measures. Only directly reported or reproducibly calculated estimates were retained, while HRs were analyzed separately from ORs, and non-calculable findings were synthesized narratively. Overall, the evidence indicated that vitamin D deficiency was associated with greater cardiometabolic and renal disease burden, but it did not establish causality [6-12,19-27].

In rheumatoid arthritis, evidence suggests associations between vitamin D status and metabolic syndrome, dyslipidemia, endothelial dysfunction, arterial stiffness, and other cardiovascular risk markers [6-9,21,22]. Baker et al. directly reported increased adjusted odds of hyperlipidemia and metabolic syndrome among vitamin D-deficient patients [8]. By contrast, Haque et al., Delgado-Frías et al., Caraba et al., and Lo Gullo et al. primarily reported associations involving continuous biomarkers, group comparisons, or correlations that could not be converted into ORs without unsupported assumptions [6,7,9,22]. These studies supported the direction of the cardiometabolic relationship but were unsuitable for inclusion in one OR-based pooled estimate.

Rivera-Escoto et al. evaluated calcidiol, calcitriol, the calcitriol-to-calcidiol hydroxylation-efficiency ratio, and soluble vitamin D receptor concentrations in relation to cardiovascular risk [21]. Although ORs were reported, the principal exposure was elevated calcitriol or hydroxylation efficiency rather than vitamin D deficiency defined exclusively by serum 25(OH)D. These estimates were therefore not combined with deficiency-versus-sufficiency ORs. This distinction is important because pooling biologically different vitamin D measures as if they represented the same exposure would produce a clinically misleading summary estimate [21].

Studies involving SLE and juvenile dermatomyositis associated lower serum 25(OH)D concentrations with disease activity, proteinuria, vascular stiffness, metabolic syndrome, and insulin resistance [10-12,20,23-26]. Robinson et al. identified a relationship between severe vitamin D deficiency and renal involvement in pediatric disease. Still, they reported that an OR could not be calculated due to the observed distribution of the data [10]. Attar and Siddiqui primarily examined the prevalence and clinical correlates of vitamin D deficiency rather than reporting a directly comparable OR for renal events [23]. These findings were therefore retained in the qualitative synthesis rather than being assigned reconstructed effect estimates unsupported by the source data.

Pediatric findings require separate interpretation because disease duration, treatment exposure, developmental characteristics, and the timing of cardiovascular and renal complications differ from those in adults [10,20]. Additionally, proteinuria may contribute to low circulating vitamin D through urinary loss of vitamin D-binding protein, creating the possibility of reverse causation. The relationship between vitamin D deficiency and renal involvement in pediatric SLE may consequently reflect both the effect of active renal disease on vitamin D status and the potential biological effects of deficiency [10,20].

Evidence concerning Sjögren syndrome was limited. Zardi et al. reported relationships between vitamin D status and disease activity or accumulated damage but did not establish an independent association with subclinical atherosclerosis [27]. Chen et al. reported increased hazards of mortality, major adverse cardiovascular events, and major adverse kidney events among patients with vitamin D deficiency [19]. Because these were time-to-event HRs from a single retrospective cohort, they were retained as HRs rather than converted to ORs or combined with cross-sectional renal outcomes. The three endpoints were also not treated as three independent studies, preventing artificial inflation of precision [19].

The observed relationships are biologically plausible. Vitamin D contributes to immune tolerance, T-cell differentiation, B-cell regulation, and inflammatory cytokine signaling [13,14]. Deficiency may coexist with systemic inflammation, oxidative stress, insulin resistance, endothelial injury, and altered activity of the renin-angiotensin-aldosterone system [5,15]. Nevertheless, these mechanisms do not prove causality. Active disease, photosensitivity, limited sunlight exposure, obesity, reduced physical activity, glucocorticoid treatment, and renal dysfunction can reduce vitamin D concentrations while independently increasing cardiometabolic and renal risk [1,16].

Serum 25(OH)D measurement may contribute to nutritional and skeletal assessment in patients with ARDs. However, it should not be used as an isolated prognostic test for cardiovascular or renal outcomes. Vitamin D status should be interpreted alongside disease activity, conventional cardiovascular risk factors, renal function, obesity, treatment exposure, season, sunlight exposure, and comorbidities [1,3,15,16]. Although correction of confirmed vitamin D deficiency remains appropriate for established skeletal and mineral indications, current interventional evidence does not demonstrate that supplementation prevents cardiovascular events, kidney events, or mortality in these disorders [17,18].

A principal strength of this review was the source-level verification of quantitative estimates, which prevented incompatible biomarkers and effect measures from being combined. The primary limitations were the observational and predominantly cross-sectional designs, small or single-center samples, heterogeneous vitamin D assays and thresholds, inconsistent outcome definitions, surrogate endpoints, and variable adjustment for confounding [6-12,19-27]. The small number of independent and statistically compatible estimates restricted outcome-specific pooling, subgroup analyses, meta-regression, and reliable assessment of publication bias. Combining adult and pediatric evidence also introduced clinical indirectness [10,20].

The certainty of the evidence was low due to risk of bias, inconsistency, indirectness, imprecision, and residual confounding. Although the direction of several associations suggested that vitamin D deficiency may identify patients with greater systemic disease burden, the evidence was insufficient to establish vitamin D deficiency as an independent cause or validated predictor of cardiovascular and renal events. Certainty should therefore be evaluated separately for metabolic, vascular, renal, cardiovascular, and mortality outcomes [6-12,19-27].

Future studies should use prospective multicenter designs, standardized serum 25(OH)D thresholds, repeated measurements, and clearly defined cardiovascular and renal endpoints [28,29]. Analyses should account for season, sunlight exposure, obesity, inflammatory activity, renal function, glucocorticoid dose, immunosuppressive therapy, and vitamin D supplementation. Adult and pediatric populations should be examined separately. Randomized controlled trials are required to determine whether correcting confirmed vitamin D deficiency improves disease activity or reduces cardiovascular, renal, and mortality outcomes [17,18].

## Conclusions

Vitamin D deficiency was associated with greater cardiometabolic and renal disease burden across ARDs. The most consistent findings involved metabolic and vascular abnormalities in rheumatoid arthritis, disease activity and renal involvement in SLE and juvenile dermatomyositis, and cardiorenal events in Sjögren syndrome. However, heterogeneous exposures, outcomes, and effect measures prevented a defensible conclusion of a nearly twofold increase in risk. Given the observational evidence and potential residual confounding, vitamin D deficiency should be considered a possible marker rather than a proven cause of cardiovascular or renal vulnerability. Prospective studies and randomized controlled trials are needed to determine whether correcting deficiency improves long-term clinical outcomes.
